# Ultrasensitive photodetectors exploiting electrostatic trapping and percolation transport

**DOI:** 10.1038/ncomms11924

**Published:** 2016-06-21

**Authors:** Yingjie Zhang, Daniel J. Hellebusch, Noah D. Bronstein, Changhyun Ko, D. Frank Ogletree, Miquel Salmeron, A. Paul Alivisatos

**Affiliations:** 1 Applied Science and Technology Graduate Program, University of California, Berkeley, California 94720, USA; 2 Materials Sciences Division, Lawrence Berkeley National Laboratory, 1 Cyclotron Road, Berkeley, California 94720, USA; 3 Department of Chemical Engineering, University of California, Berkeley, California 94720, USA; 4 Kavli Energy NanoScience Institute, Berkeley, California 94720, USA; 5 Department of Chemistry, University of California, Berkeley, California 94720, USA; 6 Department of Materials Science and Engineering, University of California, Berkeley, California 94720, USA; 7 The Molecular Foundry, Lawrence Berkeley National Laboratory, Berkeley, California 94720, USA

## Abstract

The sensitivity of semiconductor photodetectors is limited by photocarrier recombination during the carrier transport process. We developed a new photoactive material that reduces recombination by physically separating hole and electron charge carriers. This material has a specific detectivity (the ability to detect small signals) of 5 × 10^17^ Jones, the highest reported in visible and infrared detectors at room temperature, and 4–5 orders of magnitude higher than that of commercial single-crystal silicon detectors. The material was fabricated by sintering chloride-capped CdTe nanocrystals into polycrystalline films, where Cl selectively segregates into grain boundaries acting as n-type dopants. Photogenerated electrons concentrate in and percolate along the grain boundaries—a network of energy valleys, while holes are confined in the grain interiors. This electrostatic field-assisted carrier separation and percolation mechanism enables an unprecedented photoconductive gain of 10^10^ *e*^*−*^ per photon, and allows for effective control of the device response speed by active carrier quenching.

## Abstract (web summary)

Photodetector sensitivity is limited by charge carrier recombination. Here, Zhang *et al*. use nanocrystal ligand engineering and sintering to provide separate pathways for electron and holes, and to fabricate photodetectors with a high detectivity, especially promising for applications in low-light imaging.

## Graphical abstract

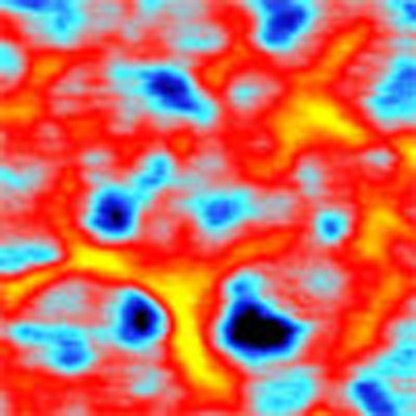

Photodetectors, devices that convert photons to electricity, are widely used in digital imaging, optical communications, remote sensing, night-time surveillance, medical imaging and so on.^1^,^2^,^3^ Their sensitivity, the ability to differentiate signal from noise, is key for high-fidelity photon detection and imaging, especially when the signal is weak. To achieve superior sensitivity, a high gain is crucial to amplify the signal far above the noise baseline. One way to accomplish this is to multiply the photogenerated charge carriers in a single carrier transport and collection cycle, as done in photomultiplier and avalanche photodiode devices, which achieve typical gains of 10^3^–10^8^ carriers per incident photon^4^,^5^. However, the required high bias (hundreds to thousands of volts) and their bulky nature restrict their integration with micro-electronics for digital imaging. In addition, the electron multiplication processes give rise to excess noise. The other approach towards high gain is to collect each photocarrier multiple times over many transport cycles in simple, two-terminal devices with semiconductor channels^3^,^6^,^7^,^8^. These devices, known as photoconductors (a subset of photodetectors), are designed to trap the minority charge carriers for a long time, enabling majority carriers to recirculate through the device many times before recombining. In this way, multiple carrier collection occurs with the absorption of one photon. Small and simple in design, photoconductors are compatible with modern micro-electronics, and can be integrated as, for example, pixel sensors in the widely used CMOS (complementary metal–oxide–semiconductor) technologies.

The performance of a photoconductor device depends critically on the deliberate control of minority carrier trapping with the goal of achieving long carrier lifetime while preserving the high-mobility, low-noise majority carrier transport. Typically, minority carriers are retained in sub-bandgap states or electrostatic barriers induced by defects, dopants, electronic junctions or a combination of these factors^1^,^6^,^7^,^8^,^9^. However, these minority carrier traps are often in the pathway of majority carrier transport, leading to carrier scattering, reduced mobility and noise, which limit the photon sensitivity.

Here we present a novel strategy to engineer a heterogeneous system with unprecedented photo-sensitivity by sequestering the majority and minority carriers in different local domains using grain boundary delta doping (GBDD). We take advantage of the discovery by Cahen and co-workers^10^,^11^ that chloride doping at grain boundaries (GBs) in CdTe polycrystalline films establishes local p–n junctions that localize electrons in the GB regions, and direct holes towards the grain interiors (GIs). We engineer the GB doping, density and connectivity by exploiting the versatile surface chemistry of colloidal nanocrystals (NCs), and fabricate a photoactive film via kinetically controlled sintering of Cl-capped CdTe NCs. The spatial separation between photogenerated electrons and holes and the defect passivation in the GBs enables low-noise electron transport and high photoconductive gain simultaneously. We further demonstrated that electrostatic quenching enables a device reset in about 100 μs, much faster than previously reported high-gain photoconductors^8^,^9^.

## Results

### Materials design and properties

**Figure 1:**
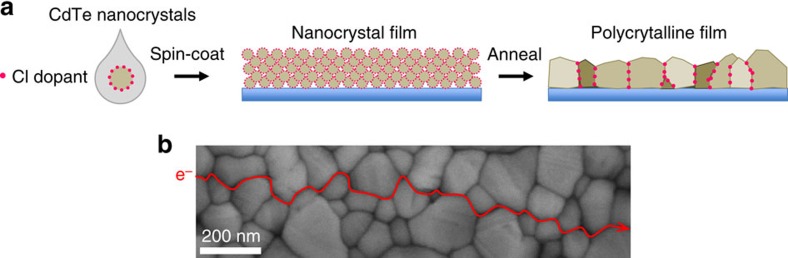
Materials design and fabrication. (**a**) Schematic of the fabrication of a polycrystalline film from colloidal CdTe nanocrystals. Chloride-capped CdTe colloidal nanocrystals were spin coated onto a substrate forming a film, which was subsequently annealed at 350 °C in an argon environment for 0.5–5 min. (**b**) Scanning electron microscopy (SEM) image of the polycrystalline film. Red line and arrow represent a pathway along GBs for electron percolation.

**Figure 2:**
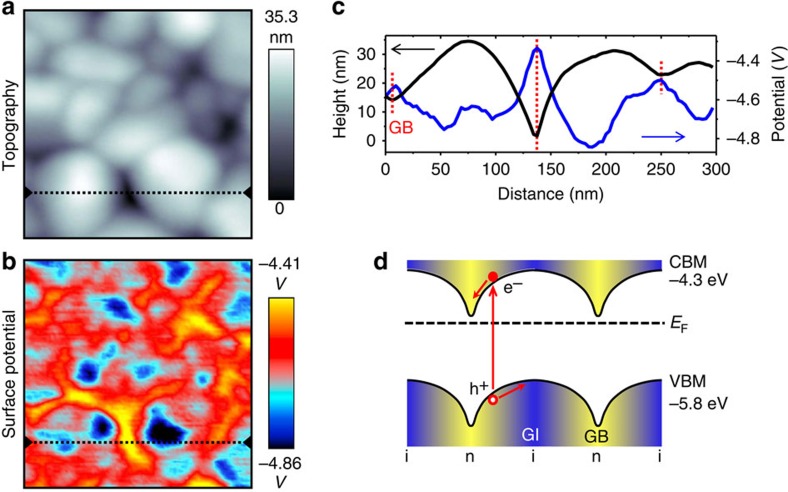
Materials properties and device design. (**a**) KPFM topography and (**b**) surface potential image obtained simultaneously on one area. Image size for **a** and **b**: 300 × 300 nm^2^ (**c**) Height and surface potential profile corresponding to the dashed lines in **a** and **b**. (**d**) Schematic of the band diagram in real space, illustrating how the photoexcited electrons slide down to the GBs while holes move towards GIs. i, intrinsic (blue regions); n, n-type (yellow regions).

In the last decade, there have been significant advances in surface modification of colloidal NCs^12^,^13^,^14^. Here we propose the new concept of GBDD to take advantage of the rich surface chemistry of NCs for bottom–up fabrication of mesoscale materials. We prepared CdTe NCs surface modified with the eventual dopant atoms, in this case Cl. We controllably annealed arrays of NCs so that they sintered into polycrystalline films, during which process Cl selectively segregates to the GBs, doping these regions n-type (Fig. 1, Supplementary Fig. 1). The engineered GBDD establish built-in electric fields making the GIs fully depleted. These fields guide hole trapping in GIs and facilitate electron percolation along the GBs (Fig. 2).

The spatial heterogeneity in doping, electrostatic potential and electronic transport—critical to the designed photodetector—are characterized by local microscopy and spectroscopy techniques. Nano-Auger electron spectroscopy confirmed that Cl is concentrated in the GBs of the sintered polycrystalline film (Supplementary Fig. 2). Local potential variations are resolved by Kelvin probe force microscopy (KPFM)^15^,^16^, revealing that the Fermi level of GBs and GIs are about −4.4–4.5 eV and −4.8–4.9 eV (relative to vacuum level), respectively (Fig. 2a–c and Supplementary Fig. 3). Since the conduction band minimum (CBM) and valence band maximum (VBM) are at approximately −4.3 eV and −5.8 eV, respectively (relative to vacuum level)^17^, we conclude that the GBs are heavily n-doped while the GIs are weakly n-doped or nearly intrinsic (Fig. 2d). Spatial current mapping using conductive atomic force microscopy (CAFM) revealed a higher conductivity in GBs compared with GIs (Supplementary Fig. 4), as expected from the higher carrier concentration and trap-passivation in the GBs. Note that a previous work showed the process of sintering Cl-capped CdSe NCs into a high-mobility film, but neither the spatial distribution of Cl nor its role in doping was identified or discussed^14^.

Our film properties contrast with those of the traditional top–down deposited CdTe polycrystalline films, which have micron scale grains^10^,^11^ significantly larger than those in our films (50–200 nm). The potential in these large grains is mostly flat with small band bending only near the GBs (about 100 mV), due to the less effective Cl doping in GBs and the small depletion region width (about 200 nm). Although used for efficient solar cells, these films are not applicable for high-gain photoconductors since photocarriers in the flat band and low-barrier GB regions are subject to rapid recombination. This is due to a lack of driving field for carrier separation in the GIs and the overlap of the transport pathways of electrons and holes (holes can tunnel through GBs), which yield short carrier lifetimes (nanoseconds to microseconds)^18^,^19^.

Our bottom–up fabricated device has several advantages compared with previous high-gain detectors: long carrier lifetime up to about 10 s, due to the long spatial separation between electrons and holes (tens of nm) and the large electrostatic potential barriers (hundreds of mV) between them; low-noise electron transport with high mobility, achieved by GBDD and trap-passivation in the GBs; tunable response time since the hole trapping electrostatic barrier can be overcome by pulsed injection of electrons leading to rapid recombination; low-cost solution-processable and scalable fabrication process, where the thickness of the film can be tuned by changing the concentration of the NC solutions and/or by repeating the spin-coating and annealing procedures multiple times.

### Electronic transport

**Figure 3:**
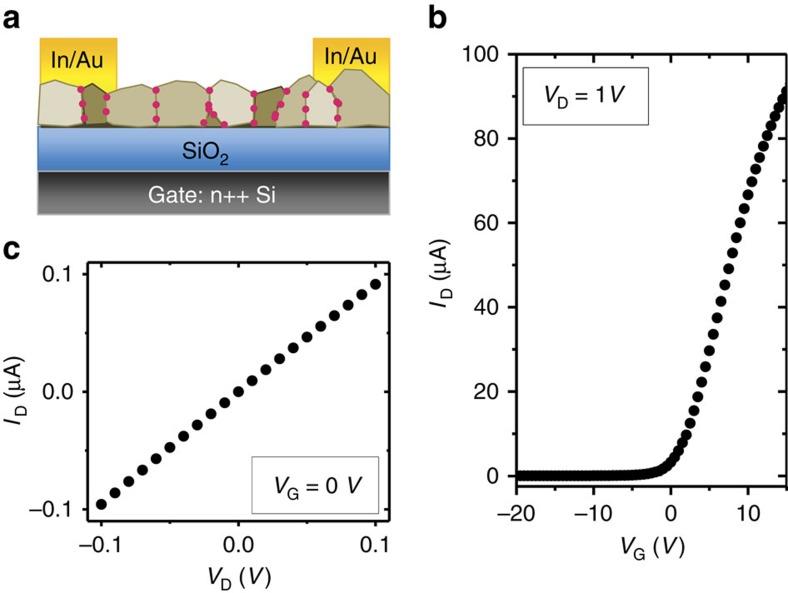
Electronic transport. (**a**) Schematic of FET structure. (**b**) Drain current (*I*_D_)–gate bias (*V*_G_) curve, with a fixed drain bias *V*_D_=1 V. From the slope of the curve at *V*_G_>0 V, we calculate the mobility of this film to be *μ*_FET_=8.9 cm^2^ V^−1^ s^−1^. (**c**) *I*_D_–*V*_D_ curve of the same device at *V*_G_=0 V, which gives a conductivity of *σ*=2.5 mS cm^−1^. FET channel width: 3 mm; length: 40 μm; thickness: 50 nm. SiO_2_ thickness: 300 nm. Measurements were performed in the dark.

Field-effect transistor (FET) measurements under dark conditions confirmed n-type electronic transport (Fig. 3). Since the GBs form a network with a large electrostatic electron confinement energy (around 0.4 eV with no or small gate bias) and a higher conductivity than the GIs, we expect the GBs to be the dominant electron transport pathways. From the gate bias-dependent current, we obtain a spatially averaged field-effect mobility of *μ*_FET_∼5.2±2.3 cm^2^ V^−1^ s^−1^ and a conductivity of *σ*∼10^−4^–5 × 10^−3^ S cm^−1^ at *V*_G_=0 V from devices made from several batches of CdTe NCs, with the best *μ*_FET_∼10 cm^2^ V^−1^ s^−1^. The actual mobility and conductivity of the GBs can be much larger since most of the volume of the material is occupied by the GIs, which are inactive for transport in the dark. Quantitative carrier concentration analysis from the KPFM and FET results further confirms that electron transport occurs mainly through GB regions (Supplementary Note 1).

We performed control studies of films sintered from CdTe NCs with no Cl or with traditional top–down Cl treatments (Supplementary Note 2), and found that they have inferior structure and much lower mobility (Supplementary Figs 5 and 6). This is strong evidence that Cl is an essential ingredient for a high-mobility film and that our bottom–up GBDD approach (incorporating Cl onto the NC surface before sintering) is key for efficient Cl doping.

### Photoconduction

**Figure 4:**
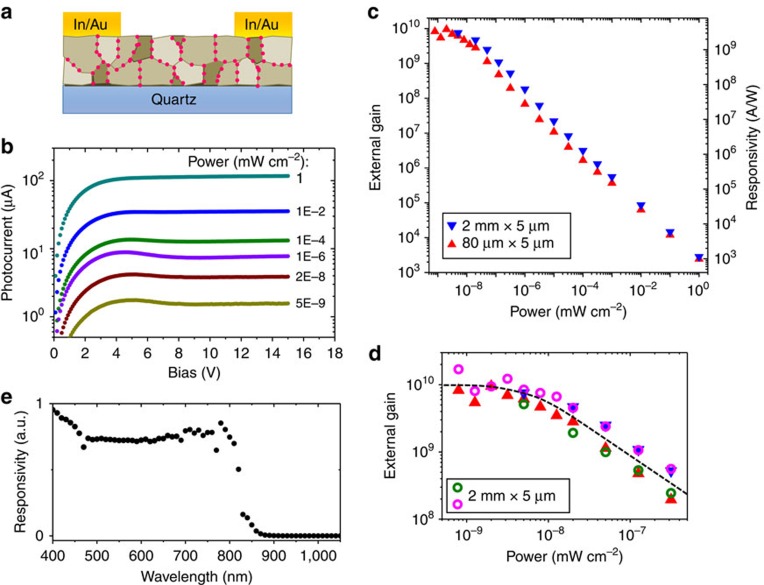
Photoconduction. (**a**) Schematic of the photoconductor device structure. (**b**) Photocurrent versus bias of a representative photoconductor, measured with 500 nm monochromic light over a range of incident power (as labelled in the figure). Channel thickness: 320 nm; length: 5 μm; width: 2 mm. Extended data on current, photocurrent and dark current is presented in Supplementary Fig. 7. (**c**) External photoconductive gain and responsivity of photoconductor devices. Incident wavelength: 500 nm. Bias: 10 V. Channel width and length are labelled in the figure, where the inverted triangle points represent the same device as that in **b**. (**d**) External gain of four devices at small photon power, where the triangle and inverted triangle points are the same as that in **c**. The dashed line is drawn to guide the eye. (**e**) Spectral response of a 2 mm × 5 μm device. The fluctuations are not due to the noise in the detector, but rather the spectral intensity variations of the light source (Supplementary Fig. 8).

To explore the photo-sensitivity of the material, we fabricated photoconductor devices based on 300–400 nm thick CdTe films (Fig. 4a). Figure 4 reports the photoresponse of two representative devices with channel widths of 2 mm and 80 μm and the same length of 5 μm. Photocurrent was generated by shining collimated, monochromatic light onto the entire channel region of the device. Our devices have a large dynamic range, sensitive to light intensities varying over 9 orders of magnitude (Fig. 4b–d, Supplementary Fig. 7). The photoconductive gain and responsivity (photocurrent divided by incident photon power) increases with the decrease of photon intensity until the power reaches 5 × 10^−9^ mW cm^−2^. This is a typical behaviour for high-gain photoconductors due to the dispersion in the activation energies of the carrier trapping sites^8^,^9^. When the power is below 5 × 10^−9^ mW cm^−2^, the external photoconductive gain (the number of collected photocarriers per incident photon) saturates at *G*_ext_≈1 × 10^10^ and the responsivity reaches *R*≈4 × 10^9^ A W^−1^ (at the wavelength of 500 nm). Both values are independent of the channel width and are the highest among all the visible and infrared photoconductors known in literature. We found that the film absorbs about 65% of the incoming light, and thus the internal gain (the number of collected carriers per absorbed photon) is also of the order of 10^10^. To understand the gain mechanism, we can approximate the internal gain as *G*_int_=*τ*_lifetime_/*τ*_transit_˙*η*_sep_, where *τ*_lifetime_ is the minority carrier lifetime, *τ*_transit_ is the transit time of the majority carrier (time to move form source to drain), and *η*_sep_ is the charge separation efficiency (the efficiency of carrier separation after photogeneration of electron–hole pairs, which we assume to be close to unity due to the strong built-in electric fields)^2^. From this equation, we can see that the gain will increase with decreasing channel length (thus shorter transit time) up until the grain size limit.

These photodetectors are responsive to photons with wavelength <850 nm, the bandgap of bulk CdTe, covering the entire visible and a small part of near-infrared spectrum (Fig. 4e). The responsivity is nearly constant over the range of 400–800 nm, a property highly desirable for visible camera applications.

### Noise analysis

**Figure 5:**
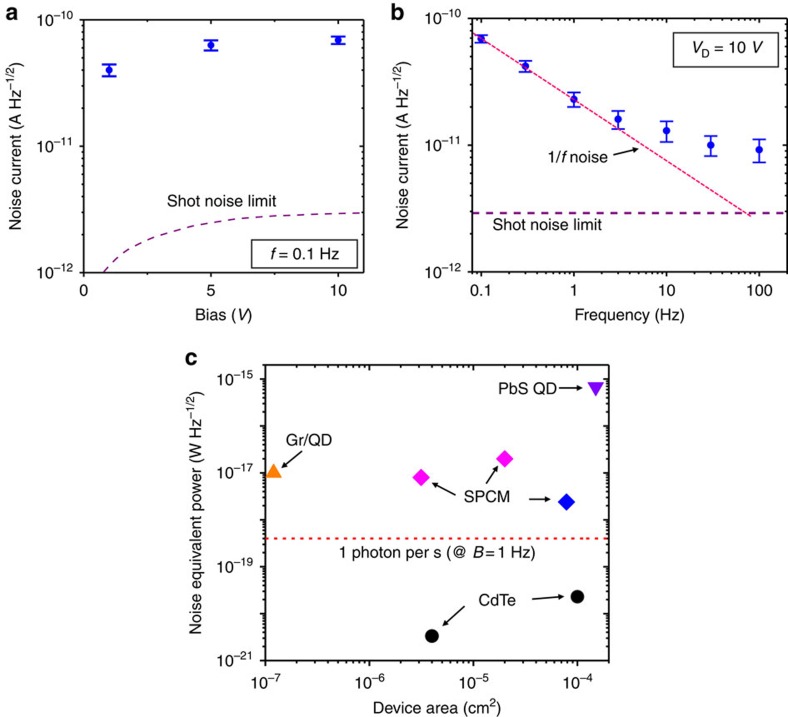
Noise and sensitivity analysis. (**a**) Noise current versus bias of a photoconductor device (2 mm × 5 μm) at a frequency of 0.1 Hz under dark condition. Error bars represent the s.d. of several measurements. Dashed line represents the shot noise limit. The results on a smaller device (80 μm × 5 μm) are shown in Supplementary Fig. 9. (**b**) Noise current versus frequency of the device at a bias of 10 V. The shot noise limit and the trend of 1/*f* noise are marked with dashed lines. (**c**) Noise equivalent power (NEP) of our two CdTe devices and other high-sensitivity devices. The values of CdTe detectors correspond to 10 V bias (with nearly the same value for 4–15 V) and 0.1 Hz frequency. Gr/QD, graphene-quantum dot hybrid^8^; PbS QD, PbS quantum dot^6^; SPCM, single-photon counting module (the most sensitive class of detectors based on inorganic crystalline semiconductors, see Methods). The dashed line represents a photon power of 1 photon per second (at a wavelength of 500 nm) when the bandwidth is normalized to 1 Hz. Details on NEP calculation of these detectors are shown in the Supplementary Note 3.

To determine the sensitivity of the device, we measured the dark noise current (*I*_noise_) and calculated the noise equivalent power (NEP), which represents the minimum detectable power at which the signal-to-noise ratio (SNR) is unity (Fig. 5) (see Methods for details on noise measurements). We found that the noise current at 0.1 Hz (the intrinsic bandwidth of the detector, inverse of the carrier lifetime, as shown later) is more than 1 order of magnitude larger than the shot noise limit, given by 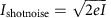, where *e* is the elementary charge. The frequency dependence of the noise reveals that the 1/*f* noise (also called flicker noise, characterized by the relation *I*_noise_^2^∝1/*f*^*α*^, where *α* is close to 1) dominates at low frequencies (*f*<10 Hz in this case), while at higher frequencies the noise current approaches the shot noise limit. This low-frequency noise has been widely observed in almost all known electronic devices^20^,^21^. Its exact mechanism is still not fully understood but it has been shown that more carrier-scattering defect sites induce higher levels of 1/*f* noise^20^,^21^,^22^. Compared with previously reported noise measurements on cadmium chalcogenide NC films and polycyrstalline films, our devices show a much less 1/*f* noise^22^,^23^, with a 1/*f* noise to shot noise ratio of about 2 orders of magnitude smaller (for *f*<10 Hz).

We calculated the bandwidth-normalized NEP using the equation NEP=*I*_noise_/*R*, where *R* is the responsivity, and *I*_noise_ was measured at a frequency of 0.1 Hz. As discussed later, we can take *R*=3 × 10^9^ A W^−1^ at 0.1 Hz and 10 V bias. This gives an NEP of 2.3 × 10^−20^ Hz^−1/2^ and 3.3 × 10^−21^ W Hz^−1/2^ for the devices with 2 mm and 80 μm channel width, respectively. Using a bandwidth of *B*=0.1 Hz, the NEP corresponds to 1 photon per 55 s (7.3 × 10^−21^ W) and 1 photon per 360 s (1.1 × 10^−21^ W), respectively, for a wavelength of 500 nm. We expect even lower NEP for smaller devices. This is an extremely high sensitivity compared with other high-performance detectors, such as PbS quantum dot photodetectors^6^, graphene-quantum dot hybrid photodetectors^8^ and commercial single-photon counting modules (SPCM) made from Si avalanche photodiodes (Fig. 5c) (see Methods for SPCM models). In cases where the photon flux is <1 photon per second, device operation speed is not a limiting factor and our detector has significant advantage over others in accurate photon sensing. However, it should be noted that lower NEP does not necessarily mean a higher SNR at all the photon power ranges. Under strong light, our device has a lower responsivity and thus may have lower SNR than photodiode-based devices.

The ultralow noise level can be attributed to the unique electronic properties of GBs and the percolation transport mechanism. It has been shown that Cl in the GBs of CdTe pushes the sub-bandgap defect states towards the VBM while moving the Fermi level close to the CBM^24^. Since the GBs in our device are heavily doped with Cl, we expect the sub-bandgap states to be filled lying far below the Fermi level and thus do not contribute to carrier trapping and scattering. This leads to a strong suppression of the 1/*f* noise that typically dominates disordered semiconductors at low frequencies (*f*<1 kHz)^21^,^22^. Another common source of noise that occurs in photodetectors under illumination is generation–recombination (g–r) noise, which originates from photocarrier trapping and detrapping processes^25^. We also expect the g–r noise to be small in our detector due to the long spatial separation between electrons and holes and the high-mobility GBs pathways for electron conduction. This is in sharp contrast with traditional polycrystalline films where the majority carriers are generated and transported through GI regions and are scattered at GBs which possess different chemical potentials than the GIs^10^,^11^.

### Temporal response

**Figure 6:**
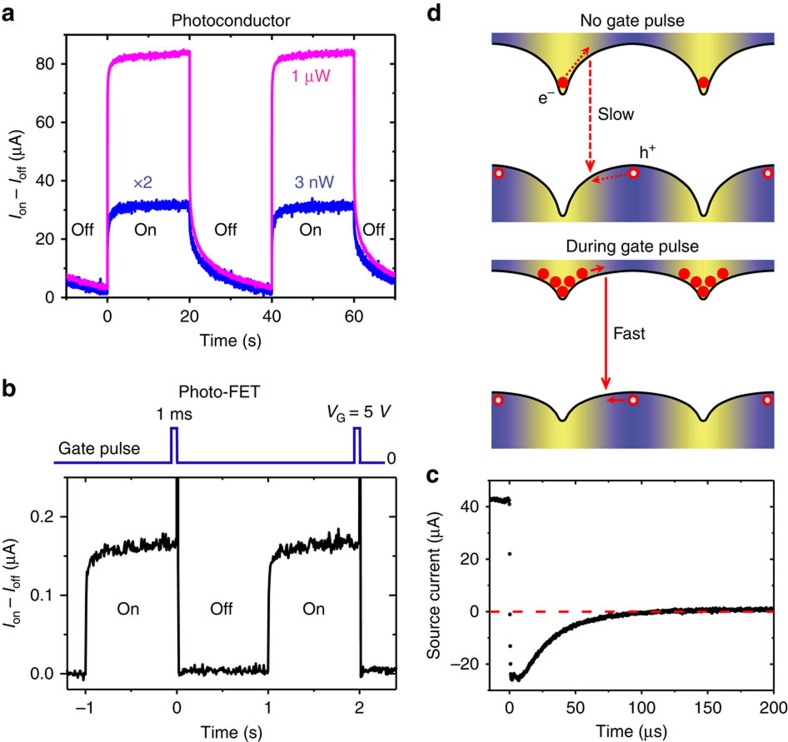
Temporal response. (**a**) Time trace of the photocurrent in a photoconductor, with the on/off modulation (interval: 20 s) of 660 nm light with two different intensities, 1 μW and 3 nW (over the channel region). Note that the photocurrent at 3 nW light is amplified by a factor of 2. Channel geometry: 2 mm (width) × 5 μm (length) × 320 nm (thickness). Bias: 1 V. (**b**) Temporal response of photocurrent in a photo-FET, in the presence of a 1 ms gate pulse synchronized with the on-to-off switch of the 660 nm light at 0.4 μW (on/off modulation interval: 1 s). Channel geometry: 3 mm (width) × 40 μm (length) × 70 nm (thickness). *V*_D_=1 V. (**c**) Transient photocurrent response (within 200 μs) of the same photo-FET device as in **b**. Note that the source current shown in **c** is the total current flowing into the source, with no dark current subtraction, while *I*_on_–*I*_off_ in **a** and **b** represents the on-state current minus the dark current. (**d**) Schematic of the band bending and carrier recombination with or without the gate pulse reset.

Besides the gain and noise level, a photodetector's temporal response also determines the extent of its applications. As previously explained, high gain in photoconductors requires long carrier lifetime, and as a result these detectors typically have slow response, in the scale of 10^−2^–10^4^ s (refs 8, 9). Figure 6a shows the time-resolved photocurrents of our photoconductor under two different light intensities. At high intensity, the photocurrent decay consists of two components: a fast decay on the scale of 1 ms, and a slow decay of ∼10 s. At lower intensity, the slow component dominates. The rise time for both power levels is in the scale of 1 ms. In the low-light case, we can see that the photocurrent decayed to ∼20% of the on-state value after the light was switched off for about 10 s (similar results were obtained for even lower photon flux). Therefore we expect the responsivity at the low-light limit to be ∼3 × 10^9^ A W^−1^ (80% of the direct current direct current (DC) value) under an AC modulation of 0.1 Hz (with a few ms on-time and 10 s off-time). We can also estimate that *τ*_lifetime_≈10 s. Taking *G*_int_≈10^10^ and assuming that *η*_sep_=1, we calculated *τ*_transit_≈1 ns, which corresponds to a mobility of ∼60 cm^2^ V^−1^ s^−1^ (Supplementary Note 4). This mobility is an order of magnitude higher than the measured bulk FET mobility, likely due to the volume averaging effect of the latter value, as mentioned above. Therefore, we expect that the actual mobility in the GBs to be in the scale of several 10 s of cm^2^ V^−1^ s^−1^. Note that in cases where *η*_sep_<1, we would expect an even higher mobility.

One strategy to improve the device response speed in high-gain detectors is to apply a gate voltage pulse^8^,^26^. Reset or quenching circuits are also widely used in photomultipliers and avalanche photodiodes. Since the hole traps in our device are electrostatic in nature, we expected that a positive gate pulse would flood the channel with electrons causing partial band flattening between the GIs and GBs, which reduces the electrostatic barrier yielding efficient electron–hole recombination (Supplementary Note 5). We employed this mechanism in a photo-FET (the same device structure as shown in Fig. 3a) and implemented a reset protocol: a 5 V gate pulse applied for 1 ms (Fig. 6b,c, Supplementary Fig. 10). The source current rapidly decreased and changed sign within 1 μs (switch time of the light), and gradually returned to the original dark current value within about 100 μs. This sign switch reveals that the source–drain transport current (electrons injected from the source to the channel and collected by the drain) decayed within 1 μs after the gate was switched off, and the injected electrons left the channel via the source and drain electrodes within 100 μs. Therefore, when the gate pulse was applied, the photocurrent decay mechanism changed from slow hole decay to fast electron decay, increasing the speed by 5 orders of magnitude. The absence of the sluggish minority carrier decay current confirms our proposed mechanism of electron–hole recombination on application of the gate pulse and is consistent with the proposed electrostatic trapping scheme (Fig. 6d). Note that this strategy is not as efficient in photoconductor devices that employ (or partially employ) sub-bandgap trap states which require higher pulse voltages to obtain only a modest improvement in response speed^8^,^9^.

## Discussion

Specific detectivity D* is a standard measure of the detector sensitivity that represents the intrinsic materials properties, independent of the device geometry^1^,^2^. We can calculate the specific detectivity 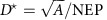 from the measured NEP and the device area *A*. In our devices, D*≈5 × 10^17^ Jones (cm Hz^1/2^ W^−1^), the highest reported for all the visible and infrared photodetectors operating at room temperature. In comparison, the specific detectivity of single-crystal silicon photodiodes is about 1 × 10^13^ Jones (ref. 27), and that of the graphene-quantum dot hybrid photodetector (one of the most sensitive photoconductor devices) is 7 × 10^13^ Jones (ref. 8).

This solution-processed, scalable detector with remarkable sensitivity and rapid response enabled by GBDD is compatible with both video-rate pixelated imaging and large-area photon detection, and should excel in applications where the detection of low levels of photons is demanded (Supplementary Notes 6 and 7). There are many situations where the photon source is naturally weak. For example, at night time the trace amounts of light from moon, star or other celestial objects can be close to or lower than 1 photon per second. Our detector, with extremely low NEP, may have advantage in this case for colour night vision^28^. There are other situations where reducing the photon exposure is highly desirable, for example, in medical X-ray detectors where X-ray generates electron–hole pairs in semiconductors and create electrical signals^29^. The much higher detectivity in our polycrystalline CdTe device compared with single-crystalline counterparts could enable significant reduction of X-ray dosage, promising for diminishing negative radiation effects on the human body during the X-ray imaging.

The GB doping and conduction properties are not unique to the CdTe system. In fact, GB-assisted photocarrier collection has been reported in a wide variety of emerging photovoltaic materials, such as Cu(In, Ga)Se_2_ (CIGS), Cu_2_ZnSnS_4_ (CZTS) and organic–inorganic halide perovskites^30^,^31^,^32^,^33^. Similar to CdTe polycrystalline films, these materials are currently fabricated mostly via top–down approaches. We expect that the bottom–up GBDD approach demonstrated here would be applicable in these systems as well. By tuning the surface chemistry of the NC building blocks and the sintering conditions, we can spatially organize the grain size, GB doping and connectivity, therefore modulating the charge transport, photoconduction and/or photovoltaic properties.

The demonstrated concept of mesoscale percolation pathway engineering can have potential impacts in other electron-based materials and devices beyond optoelectronics, involving the interaction of electrons with other degrees of freedom such as spin and phonons. As an example, in thermoelectrics we can envision a heterogeneous system where electronic transport and thermal transport take place via different pathways, and thus high electrical conductivity and low thermal conductivity can be achieved simultaneously, enhancing the thermoelectric figure of merit^34^.

## Methods

### NC synthesis

CdTe NCs capped with cadmium oleate (Cd(Oleate)_2_) with sizes of ∼5 nm were synthesized by hot injection following a procedure modified from one previously reported^35^. Specifically, 4.8 g CdO, 42 g oleic acid and 40 g octadecene were combined in a 500 ml three-neck round bottom flask. This mixture was degassed under vacuum at 100 °C for an hour. Meanwhile, two identical solutions of Te in tributylphosphine (TBP) were prepared: 1.5 g Te and 12 ml TBP were added to a 24-ml vial and heated to 220 °C until the powder dissolved and the solution became clear and yellow (in about 90 mins). Once a clear yellow solution was reached, it was cooled to room temperature. After degassing the contents of the flask, the head space was backfilled with argon and heated to 270 °C to form the cadmium oleate complex indicated by a colour change from red to clear and colourless. Once the temperature stabilized at 270 °C, the flask was quickly removed from the heating mantle and the Te/TBP solutions were swiftly and simultaneously injected through 16 gauge needles into the hot solution. On injection, the clear solution quickly changed to a dark colour. The flask was quickly cooled to room temperature by spraying air and isopropanol from a wash bottle on the flask. After the solution cooled, the flask was connected to a distillation apparatus and the volatiles were vacuum distilled at 130 °C and 55 mtorr until ∼40 ml of liquid remained. The crude product in the still pot was transferred via cannula to a Schlenk flask and stored in an argon-filled glovebox.

### NC purification

NCs were isolated and purified in an argon atmosphere. Approximately 10 ml of the crude product was added to a 50 ml centrifuge tube, filled with methyl acetate to 40 ml total volume and then centrifuged at 8,000 r.p.m. for 5 min. The clear supernatant was disposed and the formed dark coloured pellet with small amount of white content was dispersed in a small amount (3–4 ml) of pentane. Methyl acetate was added to a total volume of 50 ml creating a turbid solution which was centrifuged at 8,000 r.p.m. for 5 min. The nearly clear supernatant was disposed and the pellet was cleaned with three additional pentane/methyl acetate washing steps. By the last step, the pellet no longer contained a white residue. Finally, the pellet was dispersed in 20 ml of toluene and filtered into a clean glass vial with a 0.2 μm PTFE syringe filter. The solution was then dried under vacuum until all volatiles were removed. The dried residue was dispersed in toluene reaching a concentration of 1.5 mM determined from optical absorbance^36^.

### NC surface modification with chloride

CdTe NCs surface reaction was performed in an argon environment by adopting protocols established for CdSe-Cd(oleate)_2_ NCs^13^,^14^. It is crucial to remove the residual water from all reagents and solvents using rigorous drying methods. To begin, 4 μmol CdTe-Cd(oleate)_2_ NCs (2.7 ml of 1.5 mM NC solution) was added to a glass vial. TBP was added to the NC solution to a concentration of 500 mM. While the solution was stirred, 1.4 ml of trimethylsilychloride (TMSCl) was added (12 TMSCl molecules per nm^2^ of NC surface). After the solution was stirred for 1 h, the volatiles were removed by vacuum distillation at room temperature (a small amount of liquid remained, presumably the TBP). After distilling for 1 h, 3 ml of toluene was added to the remaining product and the vial was shaken to facilitate uniform mixing and then centrifuged at 4,000 r.p.m. for 5 min. The clear supernatant was disposed and 5 ml of toluene was added to pellet and stirred. About 147 μl octylamine (three molecules per nm^2^ of NC surface) was added and the NCs immediately dispersed. After stirring for 1 h, methyl acetate was added to flocculate the solution, which was then centrifuged at 4,000 r.p.m. for 5 min. The supernatant was then disposed. The toluene/octylamine/methyl acetate step was repeated two more times. The final pellet was dispersed in 5 ml toluene, filtered with a 0.2 μm PFTE syringe filter into a clean glass vial, and then dried under vacuum for 1 h. The dark residue was dispersed in toluene to a desired NC concentration.

### Film and device fabrication

Nanostructured CdTe polycrystalline films were formed by sintering spin-coated CdTe NC solution in an inert environment. 300 nm SiO_2_/n Si or quartz substrates were cleaned by subsequent sonication in acetone and isopropanol, then dried with filtered nitrogen and finally exposed to oxygen plasma. A drop of CdTe NC in toluene solution (typically around 0.5 mM) was deposited on the substrate and spin coated at 1,500 r.p.m. The substrate was then placed on a hotplate held at 350 °C for 30 s to 5 min, yielding a polycrystalline film with thickness of about 50–100 nm. In cases where thicker films were desired, the spin-coating and annealing procedures were repeated multiple times (layer-by-layer deposition). Thick films can also be fabricated using higher NC concentrations. FET and photoconductor devices were fabricated by thermal evaporation (using shadow masks) of 60 nm indium and 40 nm gold on the CdTe polycrystalline film deposited on SiO_2_/Si and quartz substrates, respectively. Channel length and width are determined by optical microscopy and/or atomic force microscopy on the final devices.

### Local microscopy and spectroscopy characterization of the sintered film

CdTe films were prepared on n Si substrates (with native oxide) for local imaging and spectroscopy measurements.

Zeiss Gemini Ultra-55 scanning electron microscope (SEM) was used to image the top–view and cross-section morphology of the fabricated one layer and multiple layer films. Energy-dispersive X-ray spectroscopy was performed during SEM imaging which also confirmed the existence of Cl in the CdTe film.

Nano-Auger electron spectroscopy was performed on one layer CdTe films using an Omicron system equipped with a field emission gun as an electron source. The vacuum level was 10^−10^ mbar, and the spatial resolution was ∼10 nm limited by the diameter of the focused electron beam.

Conductive AFM (CAFM) and KPFM were performed on one layer CdTe films using an Agilent AFM (with home-built feedback loop for the KPFM) in an inert atmosphere. Cr/Pt-coated tips with a spring constant of ∼0.2 N m^−1^ (from BudgetSensors) were used for CAFM, while Ti/Pt-coated tips with a spring constant of ∼2 N m^−1^ and a resonance frequency of ∼70 kHz (from Olympus) were used for KPFM. Surface potential was calibrated by setting that of the grounded Au film to −5.1 V (taking its work function to be 5.1 eV)^16^.

### Transport and photoconduction measurements

All measurements were performed under inert atmosphere. DC *I–V* curves were measured using a Keithley 2636a dual source-meter. For photocurrent measurements, a xenon lamp combined with a monochromator was used to generate monochromatic visible/near-infrared light, which was collimated and directed towards the sample. Light intensity was controlled by neutral density filters and measured using a commercial Si photodiode (FDS1010 from Thorlabs). Photocurrent was determined from the DC current under illumination after subtracting the dark DC current. Spectral responsivity measurements (Fig. 4e) were performed under a modulation of 75 Hz for different photon wavelengths.

Time-resolved photocurrent measurements (Fig. 6) were performed by generating light pulses from an LED (M660L3 from Thorlabs) using an Arduino electronics board. Short-time transient current (microseconds to milliseconds) was measured with a Femto low-noise current amplifier (DLPCA-200) and a digital oscilloscope (TDS 640 from Tektronix), while long-time current traces (milliseconds to tens of seconds) were measured using the Keithley source-meters. Gate pulses were generated and synchronized with the light pulses using the Arduino board.

### Noise measurements

Dark noise current (*I*_noise_) was measured using an SR830 lock-in amplifier from Stanford Research Systems, following a previously reported procedure^6^. An isolated DC voltage source (SIM928 from Stanford Research Systems) was used to bias the device, and a Femto low-noise current amplifier (DLPCA-200) was used to amplify the current. The output of the amplifier was directly connected to the signal input of the lock-in amplifier for noise measurements, where the internal reference frequency was manually adjusted to the desired values. The measured *I*_noise_ is given in units of A Hz^−1/2^.

To validate the accuracy of the NEP measurement technique, we performed the same measurement on the FDS1010 Si photodiode. At 5 V reverse bias, we obtained a dark current of 3.6 nA and a noise current of 5.6 × 10^−13^ A Hz^−1/2^ (with a lock-in frequency of 500 Hz). The typical dark current and noise from the manufacturer's specification are 1.05 nA and 1.5 × 10^−13^ A Hz^−1/2^, close to the values we measured. Therefore we believe the measured noise current and NEP for the CdTe detectors are also accurate.

### Commercial SPCMs for NEP comparison

Single-photon counters from Thorlabs with model number: SPCM20A, SPCM50A (magenta diamonds in Fig. 5c); COUNT BLUE series SPCM from Laser Components, module: COUNT-10B (blue diamond in Fig. 5c).

### Data availability

The data that support the findings of this study are available from the corresponding authors upon request.

## Additional information

**How to cite this article:** Zhang, Y. *et al*. Ultrasensitive photodetectors exploiting electrostatic trapping and percolation transport. *Nat. Commun.* 7:11924 doi: 10.1038/ncomms11924 (2016).

## Supplementary Material

Supplementary Information Supplementary Figures 1-10, Supplementary Notes 1-7 and Supplementary References

Peer review file
