# Supplementary material for: Ultrasensitive photodetectors exploiting electrostatic trapping and percolation transport
Source: Nat Commun. 2016 Jun 21;7:11924. doi: 10.1038/ncomms11924 (PMC4919514; doi:10.1038/ncomms11924)
Supplement: Supplementary Information — Supplementary Figures 1-10, Supplementary Notes 1-7 and Supplementary References [file ncomms11924-s1.pdf]

**Supplementary Figures**

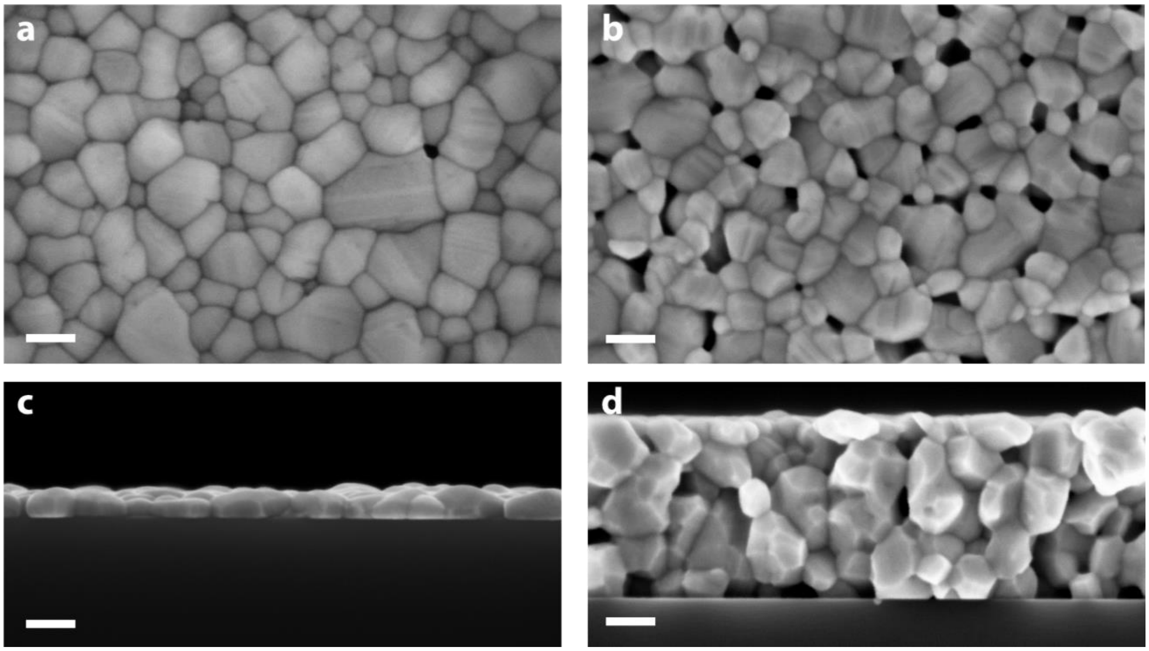


**Supplementary Figure 1. Scanning electron microscopy (SEM) images of the sintered CdTe polycrystalline films.** (**a** and **b**) top-view and (**c** and **d**) cross-section images of a ~50 nm thick film and a ~360 nm thick film, respectively. Similar grain sizes (~50 – 200 nm) are observed, revealing the scalability of the fabrication process. Scale bars: 100 nm.

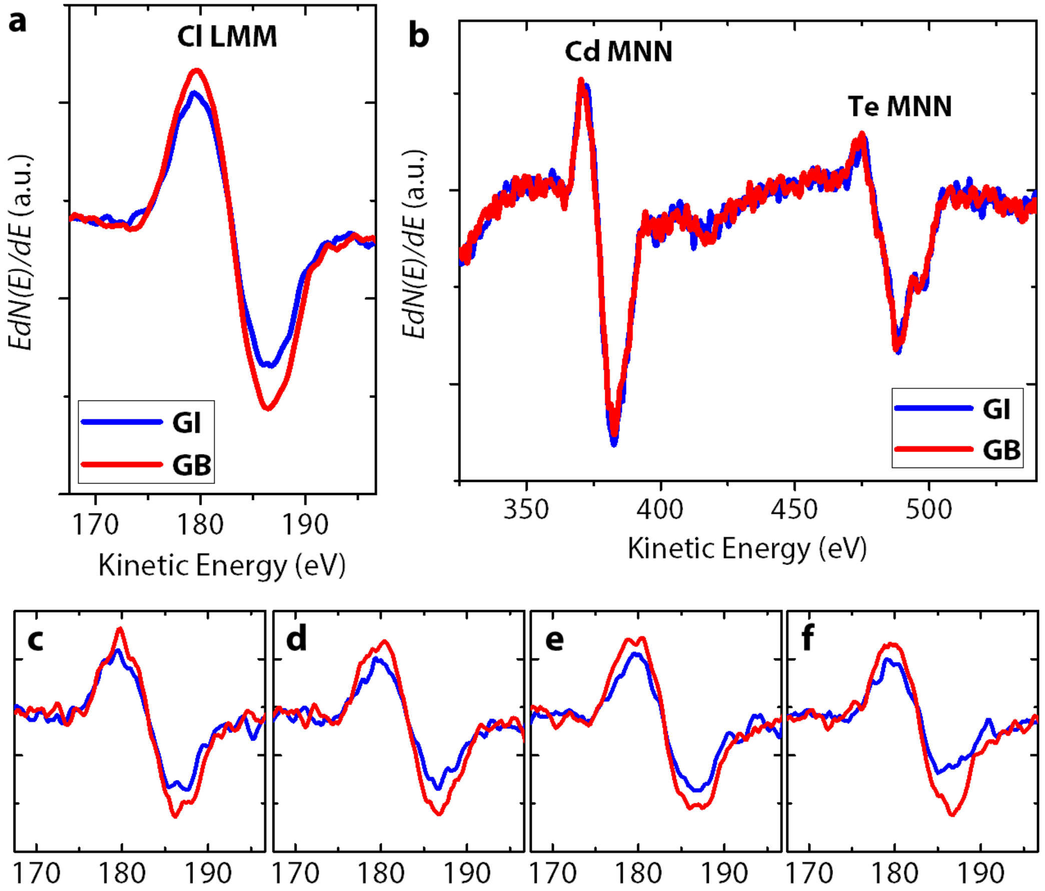


**Supplementary Figure 2. Nano-Auger electron spectroscopy of the CdTe polycrystalline film.** (**a**) Cl Auger peaks of the grain interior (GI) and grain boundary (GB) regions. Each spectrum is an average over 11 spectra from different GI or GB regions. Identical parameters were used to take the spectra at GBs and GIs. The peak-to-peak height in the spectra reveals the relative amount of Cl species. The result shows that the density of Cl species is higher in the GBs than in the GIs. Accurate quantitative values of the concentration are difficult to obtain due to the large size of the incident electron beam spot (~10 nm) compared to the GB size (~1 nm), and the possible e-beam induced evaporation of Cl. Also note that the Auger spectroscopy is sensitive to only the top ~1 nm surface region of the sample, which may not accurately represent the bulk properties. Due to these reasons, the actual ratio of the concentration of Cl in the GBs and GIs can be much larger than the ratio of the peak-to-peak height in the Auger spectra. (**b**) Cd and Te Auger peaks in a GI and a nearby GB, revealing that the concentration of these species is the same, within the noise level, in the GI and the GB (averaged over ~10 nm). Note that no peaks were observed in the spectral range of 510 – 520 nm, revealing the absence of oxygen. (**c** to **f**) Representative Cl Auger peaks at individual GI and nearby GB regions. X and Y axes are the same as those in (a).

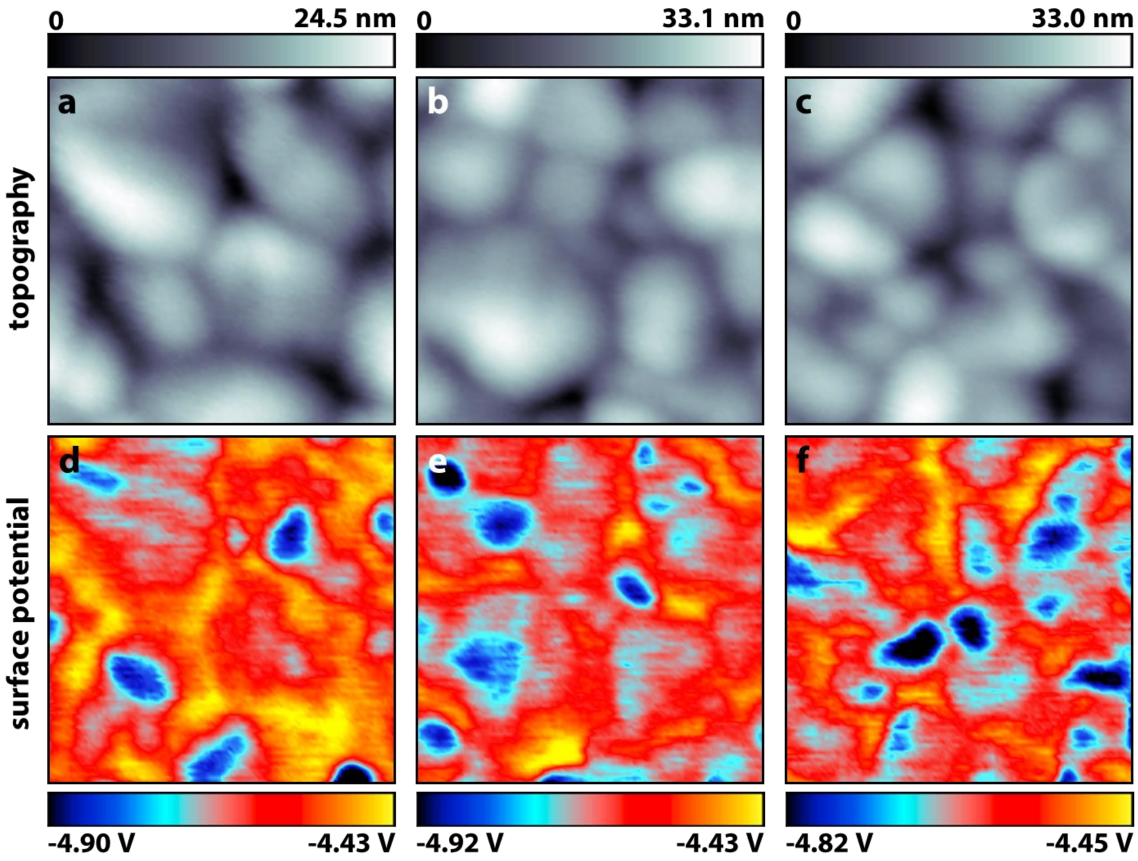


**Supplementary Figure 3. Kelvin probe force microscopy (KPFM) images of the CdTe films.** (**a** to **c**) topography images. (**d** to **f**) Simultaneously obtained surface potential images. Image size: 300 × 300 nm$^2$. GBs in general have higher surface potential (lower work function) than GIs, although the topography and potential features are not fully correlated (see also Fig. 2a–c in the main paper). This is an evidence that cross-talk artifacts are absent in our KPFM results.

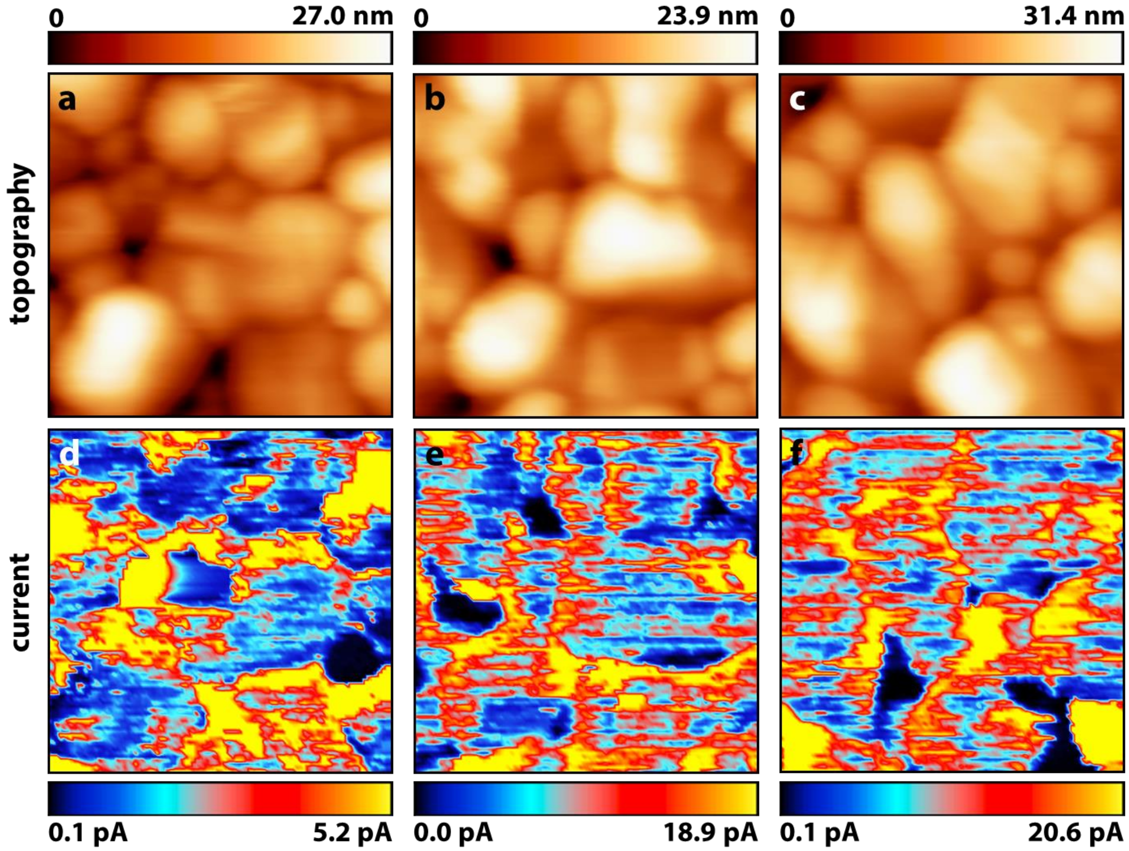


**Supplementary Figure 4. Conductive atomic force microscopy (CAFM) images.** (**a** to **c**) topography and (**d** to **f**) current images on the same areas. Image size: 300 × 300 $nm^2$. Higher current is consistently observed on the GBs compared to GIs. During the measurements the CAFM tip was in virtual ground while the sample was biased with either -3 V (a and d) or -5 V (b, e, c, f).

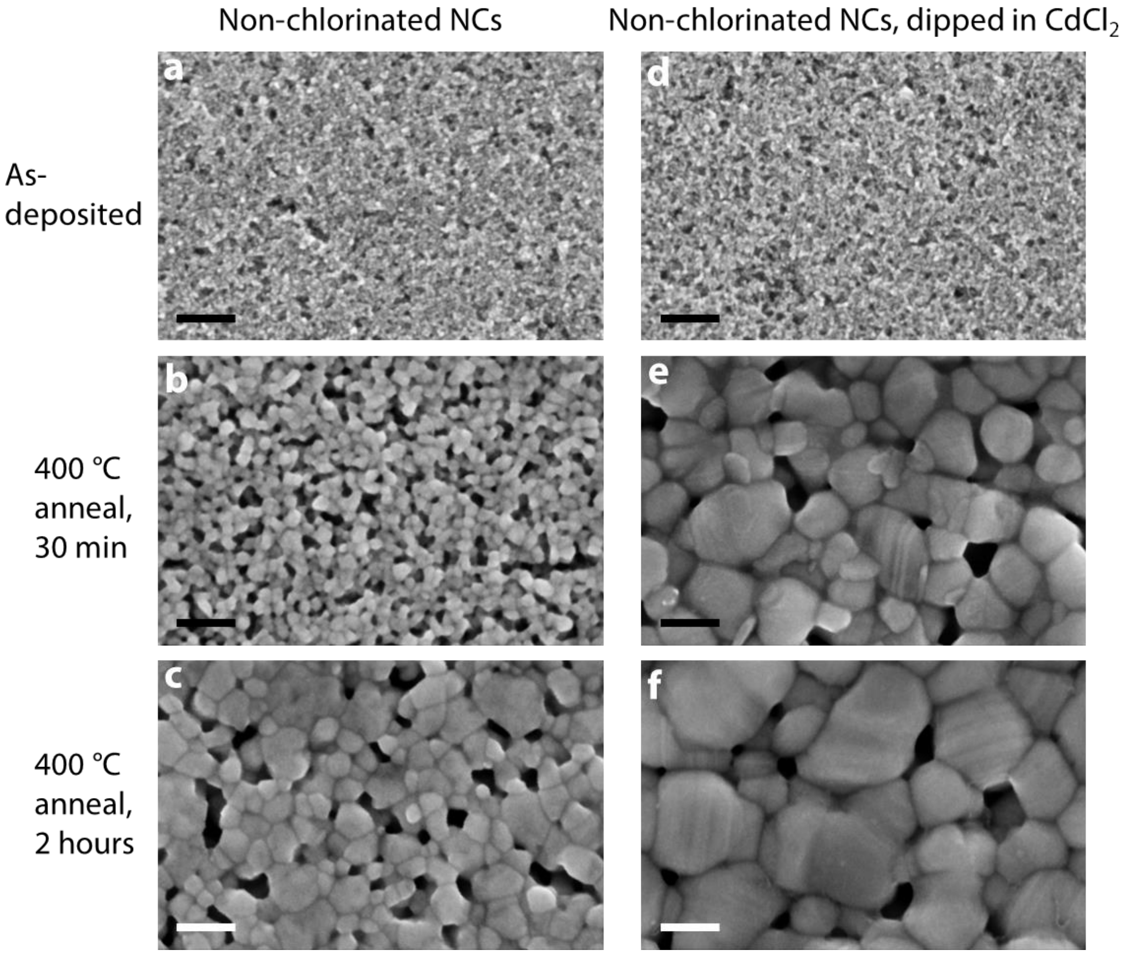


**Supplementary Figure 5. SEM images of control samples with no or different chlorine treatments.** (**a** to **c**) As deposited, 30 min annealed, and 2 hour annealed (at 400 °C) CdTe NC films where the NC has no Cl on the surface (non-Cl films). (**d**) Non-chlorinated CdTe NC films was dipped in a saturated solution of $CdCl_2$ in methanol, and (**e** and **f**) annealed at the same conditions (as the undipped sample). These are the $CdCl_2$-dip films. Scale bars: 100 nm.

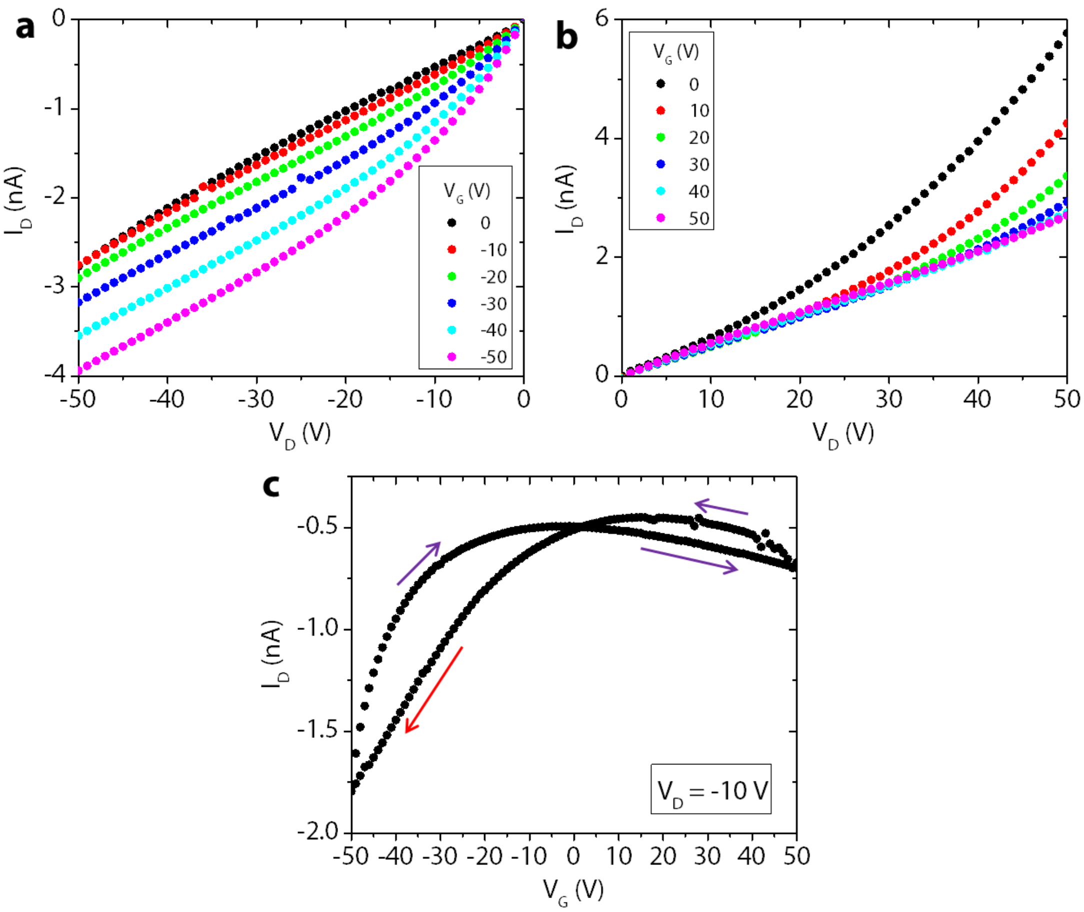


**Supplementary Figure 6. Transport measurements of a control FET device using a $CdCl_2$-dip film.** Channel width: 3 mm; length: 100 µm. The film is the same as that in Supplementary Figure 5f. (**a** and **b**) Drain current ($I_D$) – drain bias ($V_D$) curves at different gate bias ($V_G$). (**c**) $I_D$ – $V_G$ curve, where the arrows show the scan direction. A hole mobility of $1.0 \times 10^{-5}$ $cm^2/(Vs)$ is obtained from the linear regime in the scan direction shown by the red arrow.

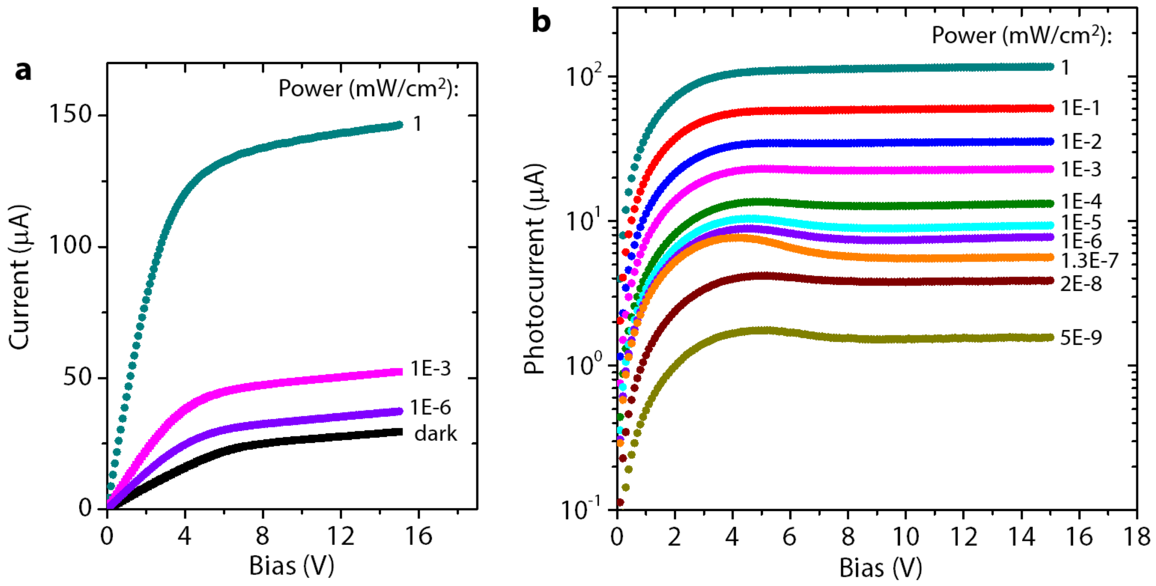


**Supplementary Figure 7. Current and photocurrent *vs* bias at different photon power.** (**a**) Raw current under dark and under monochromatic light at different power. (**b**) Photocurrent (raw current minus dark current) at a variety of photon power. Channel width: 2 mm; length: 5 µm; thickness: 320 nm. Substrate: quartz. Photon wavelength: 500 nm. Photocurrent saturates at ~4 V, and remains almost constant at higher bias. The flattening of the dark current and photocurrent at high bias is likely due to velocity saturation.

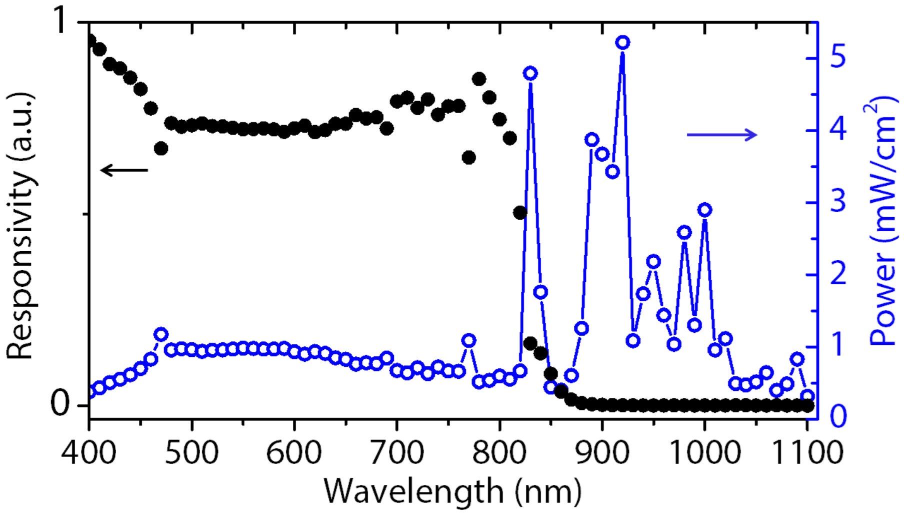


**Supplementary Figure 8. Detector responsivity and lamp power *vs* wavelength.** The responsivity spectrum is the same as the one shown in Fig. 4e in the main paper. The xenon lamp spectrum exhibits intensity fluctuations, which induces variations in the responsivity spectrum due to the non-linear intensity-dependent responsivity response of our detector (as shown in Fig. 4c in the main paper). Despite these fluctuations, the onset of the detector response at ~850 nm is clear.

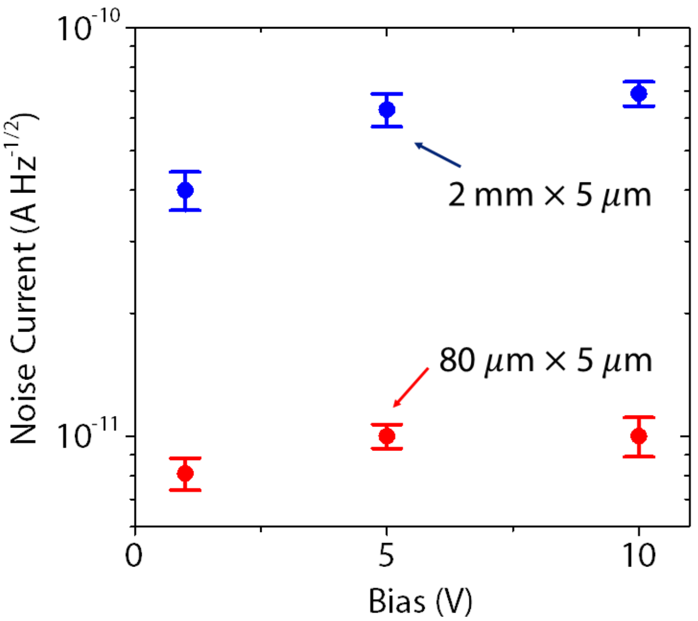


**Supplementary Figure 9. Noise current of devices with different channel width.** The results were obtained at a frequency of 0.1 Hz. The blue data points are the same as that in Fig. 5a in the main paper, and the red data points show the noise current of a smaller device as labeled. From the noise currents at 10 V of the two devices, the NEP were calculated and shown in Fig. 5c in the main paper.

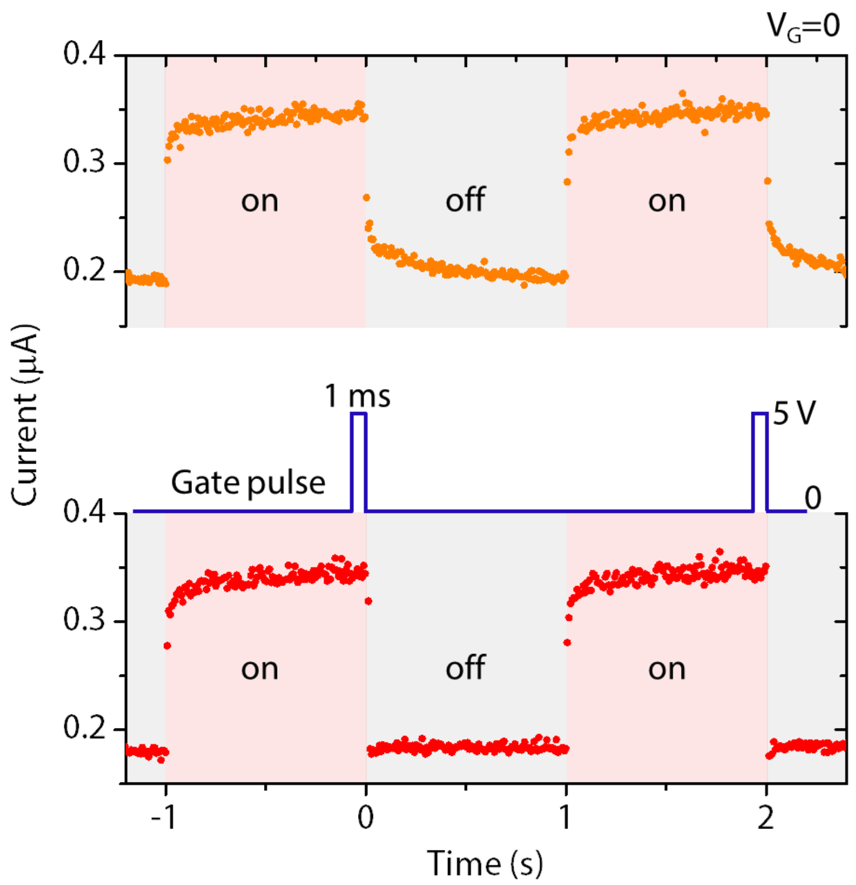


**Supplementary Figure 10. Temporal response with and without gate pulse reset.** The photo-FET device is the same as that shown in Fig. 5b. The upper panel shows the raw current under light modulation (660 nm, 0.4 µW) with zero gate bias, while the bottom panel shows the raw current under the same light modulation with a synchronized gate pulse (same as Fig. 6b except that no dark current subtraction was applied in this case). These results show that the reset function effectively accelerates the photocurrent decay while preserving its magnitude. Therefore the device can operate much faster without losing the gain and sensitivity. Note that the source current shown in Fig. 6c (in the main paper) is much higher than the raw current shown here. This is due to the 5 V gate pulse applied within 1 ms before the light was switched off. This gate pulse induces electron injection into the channel and thus a large source-drain current that is only observable at the sub-millisecond time scale, which is beyond the time resolution of the results shown in this figure.

## Supplementary Notes

**Supplementary Note 1.** Carrier concentration analysis

Carrier concentration can be calculated and compared from the KPFM and FET transport results. From KPFM results, we found that the Fermi levels ($E_F$) at GBs and GIs are ~0.1 – 0.2 eV and ~0.5 – 0.6 eV below the CBM ($E_c$), respectively. Taking the conduction band effective density of states (DOS) to be $n_c = 8 \times 10^{17}$ $cm^{-3}$ (ref. 1), we can calculate the electron carrier density at the GBs and GIs from $n = n_c \exp(-\frac{E_c - E_F}{kT})$ (ref. 2). This gives electron carrier concentration in the range of $10^{14} - 10^{17}$ $cm^{-3}$ and $10^7 - 10^{10}$ $cm^{-3}$ for GBs and GIs, respectively.

From FET transport results, we can determine the carrier concentration from $n = \frac{\sigma}{e\mu_{FET}}$ ($e$ is the absolute value of the elementary charge). Although the conductivity and mobility calculations are subject to volume averaging effects due to the nature of the percolation transport, we expect the calculated carrier concentration to be a good estimate of the carrier density in the conduction pathways since the volume factors from $\sigma$ and $\mu_{FET}$ cancel out. In this way we obtain $n$ ~ $10^{14}$ – $5\times10^{15}$ $cm^{-3}$. The carrier density estimated from the FET results agree well with that of the grain boundaries estimated from KPFM results, which is another evidence that electrons concentrate in and conduct through the GBs.

**Supplementary Note 2.** Control experiments to reveal the importance of GBDD with Cl

Our photo-sensitive CdTe polycrystalline films were made via a bottom-up approach, from surface-modified nanocrystals. The surface modification of Cl is crucial for GBDD and for the device performance. To show the advantage of this bottom-up GBDD approach (we name the sample as GBDD films for comparison), we fabricated control samples and devices using two other methods: 1. Directly sinter non-chlorinated CdTe nanocrystals to form a polycrystalline film (non-Cl film); 2. Dip a non-chlorinated CdTe nanocrystal film in a saturated solution of $CdCl_2$ in methanol at 60 °C, and then sinter the nanocrystals into a polycrystalline film ($CdCl_2$-dip film). While method 1 leads to a film with no Cl dopants, method 2 is a "top-down" approach of Cl doping which has been widely used in the process of CdTe solar cell fabrication[3]. The SEM images of the resulting films are shown in Supplementary Figure 5. We found that the non-Cl films are worse in quality and have much smaller grain sizes compared to $CdCl_2$-dip films. This is in agreement with the mechanism that Cl favors crystalline growth of CdTe, as have been widely observed in the CdTe photovoltaics community[3-6]. Comparing method 2 with our bottom-up GBDD approach (sintering chlorinated CdTe nanocrystals), we found that the former requires higher sintering temperature and/or longer sintering time, and results in a smaller Cl concentration in the film. We performed energy-dispersive X-ray spectroscopy (EDS) and found clear Cl peaks in GBDD films corresponding to an elemental concentration of ~5%. In contrast, the EDS signal of Cl in $CdCl_2$-dip films is within the noise baseline. X-ray photoelectron spectroscopy (XPS) reveals a Cl concentration of

~0.9% in a typical $CdCl_2$-dip film. These results confirm that our bottom-up GBDD approach is superior to traditional top-down methods in effective doping of Cl in polycrystalline CdTe films.

Besides structural and elemental analysis, we further performed electrical transport measurements of $CdCl_2$-dip films. We found that these films have poor conductivity and low mobility. A typical result is shown in Supplementary Figure 6. This film exhibits negligible electron mobility and a hole mobility of only ~$1.0 \times 10^{-5}$ $cm^2/(Vs)$, almost six orders of magnitude smaller than the (spatially averaged) electron mobility in our GBDD films. This is further proof that Cl passivates defects in CdTe leading to higher mobility.

**Supplementary Note 3.** NEP calculation of referenced devices

For the Gr/QD device[7], the resistance and the noise resistance was shown to be 13.6 $k\Omega$ and 0.1 $\Omega/\sqrt{\text{Hz}}$, respectively. The noise current (at 1 V bias) can be obtained as $5.4 \times 10^{-10}$ $\text{A}/\sqrt{\text{Hz}}$. Given that the responsivity is $5 \times 10^7$ $\text{A}/\sqrt{\text{Hz}}$ (for a wavelength of 600 nm), we obtain an NEP of $1 \times 10^{-17}$ $\text{W}/\sqrt{\text{Hz}}$. The device area was estimated from the optical microscopy image to be $1.2 \times 10^{-7}$ $\text{cm}^2$

For SPCM, NEP can be calculated as $NEP = \frac{h\nu}{\eta}\sqrt{2D}$, where $h\nu$ is the energy of one photon, $\eta$ is the detection efficiency, and $D$ is the dark count rate[8]. From the specification of the SPCM from Thorlabs, we have $D = 25$ Hz and 150 Hz for a detector diameter of 20 µm and 50 µm, respectively, and $\eta = 35\%$ (for a wavelength of 500 nm). These give NEP of $8 \times 10^{-18}$ $\text{W}/\sqrt{\text{Hz}}$ and $2 \times 10^{-17}$ $\text{W}/\sqrt{\text{Hz}}$, for the 20 µm and 50 µm devices, respectively. For the SPCM from Laser Components, we have $D = 10$ Hz, an active detector diameter of 100 µm, and $\eta = 70\%$ (for a wavelength of 532 nm); The calculated NEP is $2.4 \times 10^{-18}$ $\text{W}/\sqrt{\text{Hz}}$.

In the case of PbS QD detectors[9], the detector area is $A = 1.5 \times 10^{-4}$ $\text{cm}^2$, and the maximum detectivity is $D^\star = 1.8 \times 10^{13}$ Jones for a wavelength of 1.3 µm and a modulation frequency of 10 Hz. Thus we have $NEP = \sqrt{A}/D^\star = 6.8 \times 10^{-16}$ $\text{W}/\sqrt{\text{Hz}}$.

**Supplementary Note 4.** Mobility calculation from transit time

While from FET data we can only obtain spatially averaged mobility, using transit time we can extract the actual mobility of the electron conduction pathways. The mobility by definition is $\mu = v/E$, where $v$ is the (unsaturated) drift velocity and $E$ is the electric field. The velocity can be calculated from the transit time as $v = L/\tau_{transit}$, where $L$=5 µm is the channel length. At the threshold voltage for velocity saturation $V_{\text{Bias}} = 4$ V (see Supplementary Figure 7), the transit time can be extracted from the internal gain as $\tau_{\text{transit}}$ = 1 ns (see main text), while the electric field can be calculated as $E = V_{bias}/L$. Therefore we have $\mu = L^2/(V_{bias} \cdot \tau_{transit})$ = 62.5 $cm^2/(Vs)$.

**Supplementary Note 5.** Calculation of gate bias induced electrostatic barrier reduction

Here we present a simple model to estimate the decrease of the electrostatic trapping barrier in the GIs induced by the application of a gate bias $V_G$=5 V. Given that the capacitance per unit area of the 300 nm thick $SiO_2$ is $C_{ox}=1.15\times10^{-8}$ F/cm$^2$, we can obtain the injected carrier density (per unit area) $\sigma=C_{ox}V_G/e=3.6\times10^{11}$ cm$^{-2}$. Using an average grain size of d≈100 nm, we obtain an average number of electrons per grain $n_{Gr}=\sigma d^2\approx36$. Assuming that all the electrons are located at the GB regions, and considering that each GB is surrounded by two grains, each hole in the center of the grain interacts with $2n_{Gr}$=72 electrons in the nearest GBs that are r≈50 nm away. Therefore the Coulomb interaction energy is $E_{\text{Coulomb}} = 2n_{\text{Gr}} \frac{1}{4\pi\epsilon_0\epsilon}\frac{e^2}{r}$ ≈190 meV. Here $\epsilon_0$ is the vacuum permittivity and $\epsilon$=11 is the dielectric constant of CdTe[10]. This estimated Coulomb energy between the holes in GI centers and nearby electrons in GBs (induced by the 5 V gate bias) is about half of the original band bending between the GI and GB. We thus expect the gate induced electron flooding in the channel to cause significant band flattening. Note that this simple calculation assumes that electrons are all localized in GBs, which is not strictly valid since the injected electrons in GBs will repel each other and diffuse towards the GI regions. This spreading out of the electrons is also responsible for rapid electron-hole recombination. The exact value of the barrier reduction and carrier distribution is beyond the scope of this discussion.

**Supplementary Note 6.** Examples of potential applications

(1) Visible camera applications. Our CdTe detector, compatible with CCD and CMOS technologies, has a high signal-to-noise ratio and a good response speed (~1 ms with gate pulse reset) capable of video-rate imaging. Therefore it has great potential for pixelated imaging in visible cameras. In the currently dominated digital camera technology, the active pixel layer is made of single crystal silicon with a thickness of a few microns. In our CdTe detector we found that a ~300 nm thick film is capable of absorbing around two thirds of the visible light. Therefore our CdTe polycrystalline layer can potentially replace Si as the pixel layer, with the advantage that the smaller thickness will reduce pixel crosstalk. The low-cost solution processing and the ease of integration with the CCD and CMOS integrated circuits are appealing for this application.

(2) Night-vision cameras. Currently there are two types of commercial night-vision cameras: thermal imaging cameras and image intensifiers. A thermal imaging camera detects infrared light emitted by objects, and a contrast is formed if an object has a different temperature from its surroundings. This is effective in identifying warm objects in cool environments when little or no visible light is present. However, these cameras cannot directly resolve the color of the objects, losing an important feature for object identification. Moreover, objects with similar temperature cannot be distinguished. An image intensifier camera multiplies the light emitted by faint objects but requires high power, and has limited light amplification and signal-to-noise ratio. Our CdTe detector, with unprecedented sensitivity, should be capable of resolving objects under extremely low light conditions, such as the light from the moon and stars. The visible light can thus be detected to obtain images with true color. In the range of 700 – 850 nm, we can also detect a small part of the near-infrared light that provides an additional imaging channel.

A comparison of the night time illuminance level with the NEP of our photodetector reveals the advantage of the detector in night vision. The total starlight at overcast night can be as low as $3 \times 10^{-5} - 1 \times 10^{-4}$ lux, corresponding to a photon intensity of $4 \times 10^{-12} - 1.5 \times 10^{-11}\ \mathrm{W/cm^2}$ at a wavelength of 555 nm (ref. 11). Using a camera where each pixel has an area of $2 \times 2\ \mu\mathrm{m}^2$, the equivalent photon flux that needs to be detected per pixel is $2 \times 10^{-19} - 6 \times 10^{-19}$ W (~1 photon per second). As we can see from Fig. 5c, our CdTe photodetector has significant advantage over other detectors in detecting this low level of light.

(3) X-ray and Gamma ray detectors. Single crystal CdTe diodes have already been used as commercial detectors at these very short wavelengths, since CdTe has high stopping powers for X-ray and Gamma rays (see for example: X-Ray & Gamma Ray Detector from Amptek, model number: XR-100T-CdTe). To detect these high energy photons a thick device (~1 mm) is required. Our polycrystalline CdTe film can be scaled to this thickness by, for example, spray coating of nanocrystals before sintering. The high sensitivity of the detector would make it possible to image low levels of X-ray, which is important for medical applications where low X-ray dosage is desired.

(4) Cherenkov particle detection. Cherenkov radiation is an important feature revealing the speed of charged high-energy particles[12]. From the radiation position and angle the speed and nature of the particle could be tracked down. Currently Cherenkov detectors used in particle detection experiments are in the form of photomultiplier tubes (PMTs), which are expensive and demand a huge amount of power to operate, especially since thousands of the meter-sized PMTs are needed to detect trace amounts of particles. Since our detector has the highest detectivity to date and can be scaled to very large sizes due to the solution processability, we expect it to have potential impacts in the area of particle detection.

**Supplementary Note 7.** Practicalities of device integration with CMOS circuits

As we mentioned in the main text, our CdTe photodetector has a simple and small structure that is compatible with CMOS technologies. A potential challenge in the fabrication process is the 350 °C annealing process required to sinter the CdTe nanocrystals into polycrystalline films. This may induce diffusion and degradation of the metal interconnects in CMOS integrated circuits. To solve this potential problem, we can take advantage of the film transfer techniques widely used in various nanomaterials (*e.g.* nanowires and 2-dimensional materials)[13,14]. Since our CdTe polycrystalline films are solution processed (therefore the film-substrate adhesion force is low), we can prepare it on any sacrificial substrate that can survive the 350 °C annealing process, and transfer the film to another substrate (*e.g.* a CMOS circuit) either via mechanical peeling & dry transfer or selective chemical etching & wet transfer. Our substrate-independent film deposition process offers a significant advantage in this case to allow film transfer from and to various substrates.

**Supplementary References**


1. Khosroabadi, S. & Keshmiri, S. H. Design of a high efficiency ultrathin CdS/CdTe solar cell using back surface field and backside distributed Bragg reflector. *Opt. Express* **22,** A921–A929 (2014).

2. Sze, S. M. *Physics of Semiconductor Devices* (Wiley, New York, ed. 2, 1981).

3. Bosio, A., Romeo, N., Mazzamuto, S. & Canevari, V. Polycrystalline CdTe thin films for photovoltaic applications. *Prog. Cryst. Growth Charact.* **52,** 247–279 (2006).

4. Visoly-Fisher, I., Cohen, S. R. & Cahen, D. Direct evidence for grain-boundary depletion in polycrystalline CdTe from nanoscale-resolved measurements. *Appl. Phys. Lett.* **82,** 556–558 (2003).

5. Visoly-Fisher, I., Cohen, S. R., Ruzin, A. & Cahen, D. How polycrystalline devices can outperform single-crystal ones: Thin film CdTe/CdS solar cells. *Adv. Mater.* **16,** 879–883 (2004).

6. Li, C. *et al.* Grain-boundary-enhanced carrier collection in CdTe solar cells. *Phys. Rev. Lett.* **112,** 156103 (2014).

7. Konstantatos, G. *et al.* Hybrid graphene-quantum dot phototransistors with ultrahigh gain. *Nature Nanotech.* **7,** 363–368 (2012).

8. Hadfield, R. H. Single-photon detectors for optical quantum information applications. *Nature Photon.* **3,** 696–705 (2009).

9. Konstantatos, G. *et al.* Ultrasensitive solution-cast quantum dot photodetectors. *Nature* **442,** 180–183 (2006).

10. Strzalkowski, I., Joshi, S. & Crowell, C. R. Dielectric constant and its temperature dependence for GaAs, CdTe, and ZnSe. *Appl. Phys. Lett.* **28,** 350–352 (1976).

11. Rich, C. & Longcore, T. Ed. *Ecological Consequences of Artificial Night Lighting* (Island Press, Washington, DC, 2006).

12. Becker-Szendy, R. *et al.* IMB-3: A Large water Cherenkov detector for nucleon decay and neutrino interactions. *Nucl. Instrum. Meth. A* **324,** 363–382 (1993).

13. Lee, C. H., Kim, D. R. & Zheng, X. Fabricating nanowire devices on diverse substrates by simple transfer-printing methods. *Proc. Natl. Acad. Sci.* **107,** 9950–9955 (2010).

14. Bae, S. *et al.* Roll-to-roll production of 30-inch graphene films for transparent electrodes. *Nature Nanotech.* **4,** 574–578 (2010).
