# Supplementary material for: Ultrasensitive photodetectors exploiting electrostatic trapping and percolation transport
Source: Nat Commun. 2016 Jun 21;7:11924. doi: 10.1038/ncomms11924 (PMC4919514; doi:10.1038/ncomms11924)
Supplement: Peer review file [file ncomms11924-s2.pdf]

Editorial Note: this manuscript has been previously reviewed at another journal that is not operating a transparent peer review scheme.
